# Assessing the Impact of Air Pollution on Grain Yield of Winter Wheat - A Case Study in the North China Plain

**DOI:** 10.1371/journal.pone.0162655

**Published:** 2016-09-09

**Authors:** Xiuwei Liu, Hongyong Sun, Til Feike, Xiying Zhang, Liwei Shao, Suying Chen

**Affiliations:** 1 Key Laboratory of Agricultural Water Resources, The Center for Agricultural Resources Research, Institute of Genetics and Developmental Biology, The Chinese Academy of Sciences, Shijiazhuang, China; 2 Texas A&M AgriLife Research and Extension Center, Uvalde, Texas, United States of America; 3 Julius Kühn-Institut (JKI) | Federal Research Centre for Cultivated Plants, Institute for Strategies and Technology Assessment, Kleinmachnow, Germany; Tennessee State University, UNITED STATES

## Abstract

The major wheat production region of China the North China Plain (NCP) is seriously affected by air pollution. In this study, yield of winter wheat (*Triticum aestivum* L.) was analyzed with respect to the potential impact of air pollution index under conditions of optimal crop management in the NCP from 2001 to 2012. Results showed that air pollution was especially serious at the early phase of winter wheat growth significantly influencing various weather factors. However, no significant correlations were found between final grain yield and the weather factors during the early growth phase. In contrast, significant correlations were found between grain yield and total solar radiation gap, sunshine hour gap, diurnal temperature range and relative humidity during the late growing phase. To disentangle the confounding effects of various weather factors, and test the isolated effect of air pollution induced changes in incoming global solar radiation on yield under ceteris paribus conditions, crop model based scenario-analysis was conducted. The simulation results of the calibrated Agricultural Production Systems Simulator (APSIM) model indicated that a reduction in radiation by 10% might cause a yield reduction by more than 10%. Increasing incident radiation by 10% would lead to yield increases of (only) 7%, with the effects being much stronger during the late growing phase compared to the early growing phase. However, there is evidence that APSIM overestimates the effect of air pollution induced changes on radiation, as it does not consider the changes in radiative properties of solar insulation, i.e. the relative increase of diffuse over direct radiation, which may partly alleviate the negative effects of reduced total radiation by air pollution. Concluding, the present study could not detect a significantly negative effect of air pollution on wheat yields in the NCP.

## Introduction

Incoming solar radiation, the driver of plant photosynthesis, has continuously declined on the world’s land surface over the last decades [1]. This phenomenon is known as total dimming and is driven mainly by changes in cloud cover and aerosols [2, 3]. Aerosols as the most common form of air pollution reflect, absorb, and scatter solar radiation [4], causing a decrease in direct radiation and an increase in diffuse radiation, generally resulting in a reduction of total solar radiation. With the rapid economic development and expansion of industrial activities in many parts of the world, a strong increase in the concentration of anthropogenic particles in the lower atmosphere needs to be recognized [5, 6].

A global hotspot of air pollution are the densely populated coastal regions of China, which experience high levels of air pollution resulting from gas or dust emissions from transport, fossil-fuel power generation and construction activities [7, 8]. These densely populated regions are at the same time the major crop production regions of China. As such, our study region the North China Plain (NCP) produces the majority of Chinese wheat (*Triticum aestivum* L.), which is mainly cultivated as winter wheat [9]. Wheat is globally the third largest crop and an essential contributor to food security in China and the world [10].

Previous studies indicate that climate trends negatively influence wheat yields in the NCP [9, 11]. A significant increase in average temperature (primarily daily minimum temperature), a decrease in the number of sunshine hours and radiation, and a consecutive reduction in the length of the growing season are considered the respective major causative factors acting negatively on wheat yields [12, 13]. These climate trends tend to reduce the length of the growing season for winter wheat [13]. A previous study identified a reduction in incoming solar radiation of up to 5–30% in some of China’s most productive agricultural regions under hazy conditions and high aerosol concentrations [14]. For maintaining high levels of wheat productivity and its resource efficient production, it is crucial to examine how high levels of air pollution and consecutive reduction in incoming solar radiation affect winter wheat, which is cultivated during the most polluted period of the year in the NCP.

For describing air pollution the air pollution index (API) was introduced as a generalized measure, which is capable of identifying those variables that significantly affect air pollution [15]. In China, data on API is collected by the Ministry of Environmental Protection of the People’s Republic of China. In several previous studies Chinese API data was implemented to analyze the effects of air pollution on different meteorological variables such as radiation or sunshine hours [16–18]. Thus, the officially released API data from the government was used in the present study to describe the daily air pollution situation in the study region.

To the best of our knowledge, there is still no report on the interrelations of air pollution and wheat production in the NCP. Therefore, the objectives of this study are to (1) analyze the effects of API on incoming solar radiation as well as other weather factors during the growing season of winter wheat at our study site in the NCP, (2) assess the effect of potential changes in total solar radiation on wheat yields using crop model based scenario-analysis.

## Materials and Methods

A field study was conducted over 12 growing seasons at the Luancheng Experimental Station (37°53’ N and 114°41’E; elevation of 50-m) in the NCP from 2001 to 2012. The experiment field is authorized by the Chinese Academy of Sciences. Winter wheat is planted in early October and harvested in early June generally followed by the cultivation of summer maize, which forms the dominant wheat-maize double cropping system of the region. The experimental station is located in a summer monsoon climatic zone, where only about 30% of annual precipitation occurs during winter wheat growing season [19].

### Crop growth data collection

The field experiment data stems from an on-going long-term irrigation field experiment involving six irrigation treatments with four replicates. For the present study, the crop data were obtained from the 100% irrigation treatment, where plants were grown under non-limiting water conditions. Winter wheat cultivar “Shixin 733” (from 2001 to 2005) and “Kenong199” (from 2005 to 2012) were used for the 12 seasons. The two cultivars have similar yield and agronomic characteristics. Row spacing was 15-cm, and seeding rate was 300 viable seeds m^-2^. Before planting, diammonium phosphate (DAP) at 450-kg ha^-1^, urea at 150-kg ha^-1^ and potassium chloride at 150-kg ha^-1^ were broadcast and incorporated into the soil. An additional 225-kg ha^-1^ of urea was top-dressed at jointing stage in early April of each year.

### Air pollution and weather factors

Daily air pollution data were available for Shijiazhuang, which is located approximately 25 km away from the experimental site, for 2001–2012 from the Ministry of Environmental Protection of the People’s Republic of China (http://datacenter.mep.gov.cn/). Air pollution is expressed by the Air Pollution Index (API). It is calculated from the average concentrations of the principal pollutants (i.e., SO_2_, NO_2_) and inhalable particulates (PM_10_) over a 24-hour period [17]. The maximum values of IPM_10_, ISO_2_, and INO_2_ form the upper limit of the API. The factor “I” represents the 24-hr API score for the pollutant species of PM_10_, SO_2_, and NO_2_. Generally, the inhalable particulates are the dominant air pollutants in China [16, 17].

Data on daily global solar radiation (GSR), photosynthetic active radiation (PAR), ultraviolet radiation (UVR), sunshine hours (SH), minimum temperature (TMIN) and maximum temperature (TMAX), diurnal temperature range (difference between daily minimum and maximum temperature; DTR), cloud cover (CC) and relative humidity (RH) were recorded at the experimental site. Daily data on top-of-atmosphere insulation at the research site extracted from the NASA POWER Agroclimatology webpage (http://power.larc.nasa.gov/cgi-bin/cgiwrap/solar/agro.cgi) was used as proxy for potential incoming radiation.

### Simulation of winter wheat yield under different scenarios

The impact of air pollution on wheat growth and yield may be covered by the effect of inter-annual changes of more influential factors [11], therefore applying regression analysis to the twelve years’ data was not able to detect the sole effect of changes in air pollution and consequential changes in incoming GSR on grain yield. To be able to separate the confounding effects of air pollution and various weather variables, the application of crop models to simulate single factor scenarios is a viable means [10]. Hence, to assess whether air pollution induced changes of incident global solar radiation are effective on grain yield under ceteris paribus conditions, scenario-analysis was conducted using the Agricultural Production Systems Simulator (APSIM) model. The APSIM model is a cropping systems simulation model developed by the agricultural production systems research unit of Australia. APSIM has been widely tested and used in Australia, the United States, Netherlands, North Africa, and China [20]. For China, validation results of the APSIM-Wheat module were summarized in [21]. APSIM was satisfactory in simulating crop growth, yield, and water use, and could explain over 80% of the variations in crop biomass and yield in the NCP [22]. In another study conducted in the NCP showed that the simulated results explained 85% of the variations in crop yield [23]. Therefore APSIM-Wheat is considered a suitable model to simulate wheat growth and development under the climatic and soil conditions of the NCP.

Hence, in the first step APSIM-Wheat was calibrated for the local field conditions and cultivars based on the available experimental data from 2001–2012. The detailed calibration procedure followed the steps in [24]. Soil physical parameters such as soil bulk density and soil texture were kept constant over all years and simulated scenarios. Irrigation was always set as full water supply. The cultivar specific calibration of APSIM-Wheat was conducted based on the field experimental results covering the parameters listed in Table 1. Due to the high similarity of the two cultivars grown over the 12 seasons, no differentiation was implemented in the model between the two.

**Table 1:** The genetic parameters used in APSIM model for the winter wheat during the 2001–2012 seasons.

Parameters	Values
Emerg_-_to_-_endjuv (thermal time from emergence to end juvenile stage(°d)	620
Startgf_-_to_-_mat (thermal time from beginning of grain-filling to maturity(°d)	620
Potential grain filling rate (potential grain-filling rate (g per kernel per day))	0.0025
Grains per gram stem (coefficient of kernel number per stem weight at the beginning of grain-filling (g per stem))	26.5
Max grain size (potential maximum grain size (g per kernel))	0.0045
Phyllochron (phellochroninterval (°C d/leaf appearance))	85
Vern sens (sensitivity to vernalization)	1.6
Photop sens (sensitivity to photoperiod)	2.0

In the next step, the calibrated model was available to assess the effect of potential changes in radiation on crop growth. Crop simulation models including APSIM are often used to simulate crop growth and development under altered conditions of incident solar radiation, which the analysis identified as the factor strongest influenced by air pollution [25–27]. However, APSIM as well as most other common crop simulation models do not distinguish between direct radiation, diffuse radiation and UVR, but just consider the total incoming global solar radiation (GSR) as driver of photosynthesis [27]. Therefore, any potential changes in the ratio of direct and diffuse radiation and in UVR under changing air pollution could not be considered in the present analysis.

**Fig 1:**
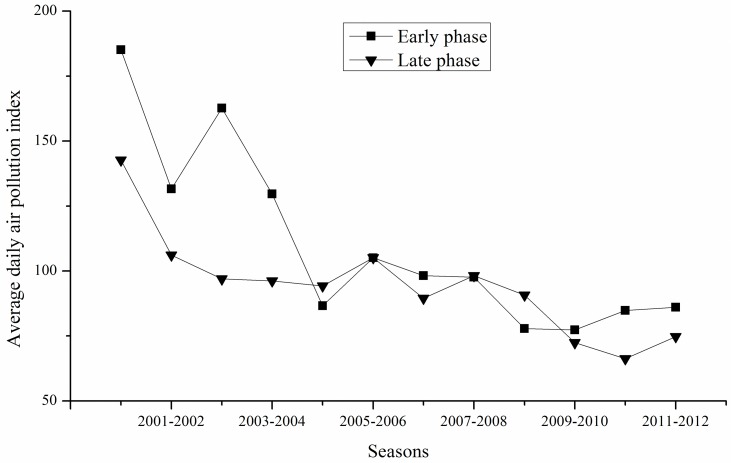
Development of average daily air pollution index during the early and late growing phases of winter wheat during 2001–2012.

To test the effect of potential changes in air pollution and related weather factors scenario-analysis was applied. The percentage reduction in radiation set up in the scenarios followed the possible effects of air pollution on total radiation. According to [16] total radiation is decreased by 8% at API-values between 100 and 200 compared to the radiation at API-values below 100. Based on the pre-analysis of API data (Fig 1), the growing period of winter wheat was separated into two growing phases: the early growing phase (from sowing to recovery after winter dormancy) and the late growing phase (from recovery to maturity). Thus, to assess the potential effects of changes in radiation on wheat yields, radiation changes were set at +/− 5% and +/− 10% for each growing phase in the scenario-analysis, which was run during 2001–2012 seasons (Table 2).

**Table 2:** Overview of the combinations of changes in daily global solar radiation (GSR) applied in the APSIM based scenario analysis.

	Change in global solar radiation [%]				
Phases	−10	−5	0	+5	+10
Early phase only	Scenario E_−10%_	ScenarioE_−5%_	CK	Scenario E_+5%_	ScenarioE_+10%_
Late phase only	Scenario L_−10%_	ScenarioL_−5%_	CK	Scenario L_+5%_	ScenarioL_+10%_
Early and late phase	Scenario EL_−10%_	ScenarioEL_−5%_	CK	Scenario EL_+5%_	ScenarioEL_+10%_

### Statistical analysis

Due to the seasonal course of the sun and its consequential effect on the daily amount of potentially incoming radiation, the daily API data could not directly be related to the ground measured incident radiation data. Hence, to assess the effect of air pollution on plant available irradiance we first calculated the daily “relative radiation gaps” for the different types of radiation as follows.

$$GSR_{gap}=\left(GSR_{p}-GSR_{i}\right)/GSR_{p}$$ (1)

where *GSR*_*gap*_ is the global solar radiation gap, *GSR*_*p*_ is the potential global solar radiation outside the atmosphere (MJ m^-2^ d^-1^) and *GSR*_*i*_ is the incident global solar radiation measured on the ground (MJ m^-2^ d^-1^).

The photosynthetic active radiation gap (*PAR*_*gap*_) was calculated as follows,

$$PAR_{gap}=\left(PAR_{p}-PAR_{i}\right)/PAR_{p}$$ (2)

where *PAR*_*p*_ is the potential photosynthetic active radiation (mol m^-2^ d^-1^) and *PAR*_*i*_ is the incident photosynthetic active radiation measured on the ground (mol m^-2^ d^-1^), with *PAR*_*p*_ derived from *GSR*_*p*_-data following the conversion recommended by [28] and recently proved viable for the region’s latitude by [29],

$$PAR_{p}=GSR_{p}*4.57/3.6$$ (3)

Accordingly, the ultra-violet radiation gap (*UVR*_*gap*_) was calculated as follows,

$$UVR_{gap}=\left(UVR_{p}-UVR_{i}\right)/UVR_{p}$$ (4)

where *UVR*_*p*_ is the potential ultra-violet radiation (MJ m^-2^ d^-1^) and *UVR*_*i*_ is the incident ultra-violet radiation measured on the ground (MJ m^-2^ d^-1^), with *UVR*_*p*_ derived from *GSR*_*o*_-data following the conversion recommended by [30],

$$UVR_{p}=UVR_{p}*0.08$$ (5)

Finally the daily sunshine hour gap (*SH*_*gap*_) was calculated as follows,

$$SH_{gap}=\left(SH_{p}-SH_{i}\right)/SH_{p}$$ (6)

where *SH*_*p*_ is the potential sunshine hours (h) and *SH*_*i*_ is the incident sunshine hours measured on the ground (h), with *SH*_*p*_ derived as follows,

$$SH_{p}=SS-SR$$ (7)

where *SS* is time of sunset and *SR* is time of sunrise.

Apart from air pollution also cloud cover (CC) reduces atmospheric transmittance. Therefore, to account for the potential effect of CC on the different radiation gaps, partial correlation was applied using daily data over the 12 investigated winter wheat seasons with the different radiation gaps, i.e., GSR_gap_, PAR_gap_, UVR_gap_, and SH_gap_ as dependent variable, API as independent variable and CC as controlling variable.

Seasonal changes and cloud cover may also affect the relation between API and other weather factors. Therefore, partial correlation analysis was applied accordingly to assess the relation of air pollution and other weather factors such as temperature and relative humidity including cloud cover and number of days after sowing as controlling variables. Finally, linear regression and correlation analyses were used to determine whether the relationships between the relative radiation gap as well as other weather variables with crop yield obtained from the field study were significant at the 95% confidence level using SPSS statistical analysis software (version 16.0).

## Results

### Effect of API on weather factors

For both growing phases the average daily API-values decreased strongly from 2001 to 2005. From 2005 onwards a continuous though only very slight decrease occurred (Fig 1). While in the first four winter wheat seasons significantly higher air pollution can be recognized during the early compared to the late growing phase, no significant differences can be recognized between the two growing phases from then onwards. Averaged over the 12 seasons the API-values for the early and the late growing phases were 110.5 and 85.6, respectively. Similarly [16] reported higher levels of air pollution in the region during winter and spring, which corresponds to the early growing phase of winter wheat.

The results of the partial correlation analysis presented in Table 3 show that during the early growing phase, all weather factors were significantly correlated with API. In contrast, no significant correlations were identified between the weather factors and API during late growing phase except for daily TMIN. Obviously air pollution significantly reduced radiation, i.e. increased the radiation gap, at early phase represented by all four radiation related variables (GSR, PAR, UVR, SH). During late growing phase no significant correlation was found between API and the radiation gaps. The difference in the effectiveness of API on weather variables during the two growing phases can largely be explained by the lower API-values during late growing phase compared to early growing phase. Additionally the contrary observations for early and late phase are a result of the difference in the angle of the sun during the two phases. At our study region (in the northern hemisphere) the angle of the sun is highest at June 21 and lowest at December 21. During early season the sun beam passes the atmosphere in a lower angle, and thus needs to pass a longer distance through the atmosphere. This results in a higher dissipation of light, and thus a higher radiation reduction by the atmosphere compared to the late season, where the angle of the sun is higher and the sun beam passes the atmosphere on a shorter distance. The significantly reduced TMIN and TMAX values due to air pollution during early phase (but not during late phase) are likely a secondary effect of reduced radiation, which leads to less solar energy (heat) reach the earth’s surface. It furthermore can be seen, that even though air pollution did not significantly affect radiation during late phase, air pollution significantly increased TMIN. This is likely a result of air pollution, which hinders the loss of reflected radiation from ground to the atmosphere leading to increased temperature, especially TMIN.

**Table 3:** Partial correlation of air pollution index with weather factors controlled for cloud cover at different growing phases of winter wheat based on daily data of 12 winter wheat seasons.

Weather factors	Early growing phase	Late growing phase
GSR_gap_	0.34**	0.14
PAR_gap_	0.26**	0.13
UVR_gap_	0.26**	0.03
SH_gap_	0.28**	0.05
TMAX	−0.52**	0.17
TMIN	−0.50**	0.24*
DTR	−0.30**	−0.08
RH	0.21*	0.11

* indicates significance at P = 0.05;

** indicates significance at P = 0.01;

GSR_gap_: relative radiation gap for global solar radiation; PAR_gap_: relative radiation gap for photosynthetic active radiation; UVR_gap_: relative radiation gap for ultraviolet radiation; SH_gap_: relative gap for sunshine hours; TMAX: maximum air temperature, TMIN: minimum air temperature, DTR: diurnal temperature range, RH: relative humidity.

During early growing phase DTR was negatively correlated to API, which is most likely a result of reduced radiation and hence reduced incoming heat energy due to air pollution during daytime. During late growing phase API has no significant negative effect on radiation and thus also no significant negative effect on DTR. Furthermore, [11] and [31] reported that DTR is additionally affected by RH and daytime temperature, which were both not affected by API in the late growing phase. While RH was significantly positively related to API during the early phase, no significant correlation occurred during late phase. There is strong evidence, that during late phase, which coincides with the beginning of rainy season, RH is mainly driven by precipitation, compared to early phase, where rainfall and evapotranspiration are much lower.

### Effect of weather changes on grain yield

**Fig 2:**
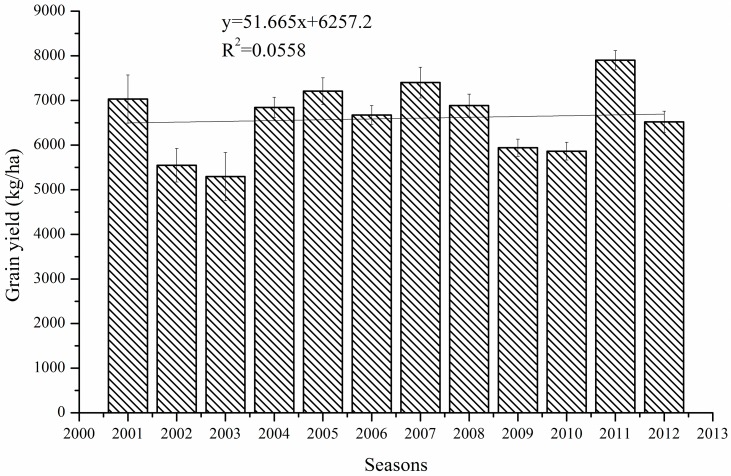
The seasonal yield variation of winter wheat from 2001 to 2012 under optimal management practices.

In the next step we assessed whether the API induced changes in weather variables are effective on grain yield. The development of winter wheat yields under well managed non-water-limited conditions during 2001 to 2012 is presented in Fig 2. During the 12 seasons large deviations from mean yield occurred ranging from −24% to +18%. As similar cultivars and identical field management practices were applied, the differences in yield level provide a good possibility to analyze the effects of different weather factors on crop production.

Correlation analyses indicated that none the weather factors, which were significantly affected by air pollution in either of the two growing phases (Table 3) showed a significant correlation with final grain yield (Table 4). On the contrary, several weather variables, which were not significantly influenced by air pollution, were significantly correlated with yield at the late growing phase, namely GSR_gap_, SH_gap_, DTR and RH. Obviously, the inter-annual differences in weather factors during the late growing phase played a more important role in the yield formation process of wheat compared to the weather conditions during early growing phase. The API measured during the late growing phase was generally lower than the values in the early growing phases and hence did not affect yield related weather factors. Consequently, an impact of air pollution induced weather factors changes on winter wheat yield could not be detected.

**Table 4:** Relationships of relative radiation gap with the final grain yield of winter wheat during the early and late growing phases during 2001–2012.

Phases	GSR_gap_	PAR_gap_	UVR_gap_	SH_gap_	TMAX	TMIN	DTR	RH
Early phase	−0.27	−0.42	0.49	−0.28	0.04	0.06	0.02	−0.45
Late phase	−0.60*	−0.48	0.22	−0.61*	0.45	0.21	0.54*	−0.66*

* indicates significance at P = 0.05;

GSR_gap_: relative radiation gap for global solar radiation, PAR_gap_: relative radiation gap for photosynthetic active radiation, UVR_gap_: relative radiation gap for ultraviolet radiation, SH_gap_: relative gap for sunshine hours, TMAX: maximum air temperature, TMIN: minimum air temperature, DTR: diurnal temperature range, RH: relative humidity.

**Fig 3:**
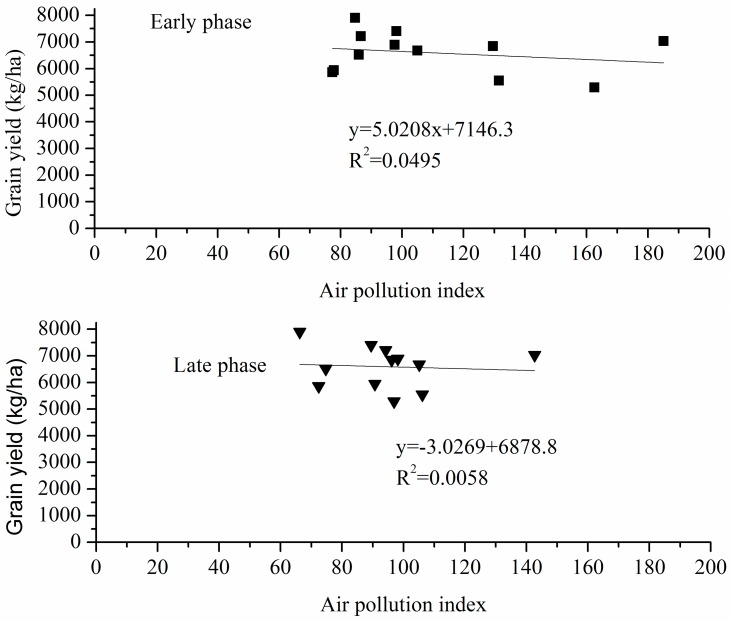
The relationships between air pollution index at different growing phases with grain yield of winter wheat.

Accordingly, also no significant correlation could be detected, when correlating the average API-values for the early and late growing phase with final wheat yield data from 2000–2012 (Fig 3). However, as mentioned above, the effect of air pollution on wheat growth and yield may just be covered by the effect of inter-annual changes of more influential factors [11, 32, 33], i.e. those weather variables showing a significant correlation with wheat yield (GSP_gap_, SH_gap_, DTR and RH during late phase).

**Fig 4:**
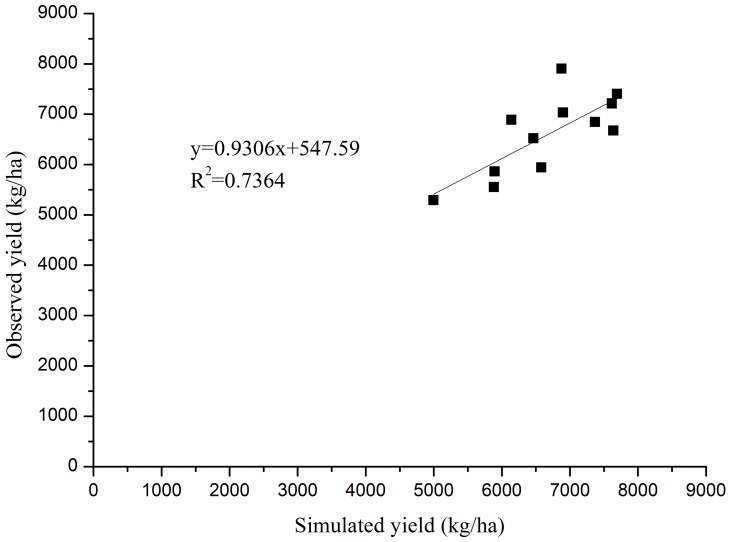
Validation plots of the APSIM-Wheat model simulation with field-observed crop yield.

Hence, crop modeling was used to assess whether air pollution induced changes of incident global solar radiation are effective on grain yield under ceteris paribus conditions. The APSIM-Wheat model was calibrated satisfactorily (Fig 4) and could be used to simulate the isolated effect of changes in GSR on grain yield. Table 5 shows the simulated grain yield under the different GSR change scenarios. Compared to the control (CK), which was simulated under the actually observed GSR-conditions, the average crop yields increased under increased GSR. Accordingly, when GSR was reduced, simulated crop yields were also reduced. For both, the GSR increase and the GSR decrease scenarios, GSR changes during early phase were less yield effective compared to GSR changes during late growing phase. This corresponds to the regression results (Table 4), where no significant yield effect of GSR_gap_ during early phase, but a significant effect during late phase was detected.

**Table 5:** Average values and standard deviations (kg/ha) of simulated wheat yields over the 12 growing seasons for different scenario combinations of increasing and decreasing radiation during early and late growing phases during 2001–2012; percentage changes compared to the observed yields under actual radiation conditions during 2001–2012 are given in brackets.

Phases	Change in global solar radiation [%]				
	−10	−5	0	+5	+10
Early phase only	6234±929	6368±952	6576±1024	6612±1005	6712±1020
	(−5.20%)	(−3.16%)	(0%)	(+0.55%)	(+2.06%)
Late phase only	5964±1013	6208±974	6576±1024	6735±990	6924±1020
	(−9.57%)	(−5.60%)	(0%)	(+2.42%)	(+5.29%)
Early and late phase	5892±934	6080±957	6576±1024	6828±1016	7064±1034
	(−10.40%)	(−7.54%)	(0%)	(+3.84%)	(+7.41%)

For all tested phases (early only, late only, early and late), the radiation reductions affected yield levels more strongly (negatively), compared to the radiation increases (positively). As such, during early phase a GSR reduction by 5% and 10% caused yield reductions of 3.16% and 5.20%, respectively, while a GSR increase by 5% and 10% caused yield increase by only 0.55% and 2.06%, respectively. Similarly, GSR reductions by 10% during the late phase and during both phases reduced grain yields by 9.57% and 10.40%, respectively, while GSR increases by 10% during the late phase and during both phases increased grain yields by only 5.29% and 7.41%, respectively. This provides evidence that the incident GSR conditions in the study region during the 2000–2012 period were already situated at the lower end of wheat radiation requirements. In accordance [11] and [23] report that GSR has been decreasing in the region over the past 30 years, which excerpts a negative effect on crop yield potential [14]. Hence, a further increase in air pollution and the consequential reduction in incident GSR may have a strong detrimental effect on wheat yields, while a reduction in air pollution and the consequential increase in incident GSR would act positive on wheat yields, however at a lower increment.

## Discussion and Conclusions

Previous research has shown that air pollution and consecutive reduction in PAR negatively affect plant growth [34, 35]. However, so far no study aimed at investigating the effect of air pollution on winter wheat yields in the air pollution hotspot North China Plain. Our analysis showed that over the 12 growing seasons air pollution was generally higher in the early growing phase compared to the late growing phase. Accordingly, air pollution (expressed by API) showed significant correlations with various weather factors including radiation, temperature and humidity, during the early but not the late growing phase. Only TMIN was positively correlated with API in the late phase. However, we could neither detect a direct correlation between API and grain yield, nor could we identify that the air pollution induced changes in weather variables exerted a significant impact on grain yield.

However, grain yield was highly associated with several weather variables at the late growing phase, which were not affected by air pollution. These factors comprise DTR and RH as well as the radiation related GSR_gap_ and SH_gap_. While higher DTR acted positively on yield, reduced radiation and increased RH were negatively related. This confirms the findings of a recent study for the same experimental site [11], which indicated that grain yield of winter wheat was positively related to DTR and SH, while it was negatively related to RH. Regarding the effect of DTR on grain yield contrasting results are obtained in previous studies. While under non-irrigated conditions increasing DTR is often negatively associated with wheat yield [36, 37], a positive correlation was identified for irrigated wheat in our study region [38]. Furthermore, [11] showed that DTR itself was affected by a combination of RH, sunshine hours, and daytime temperature, with the number of sunshine hours and the daytime maximum temperature acting positive and RH acting negative on DTR [31]. Hence, there is evidence that clear days with high TMAX and dry air lead to a high DTR, which under sufficient water supply (as investigated in our study), acts positive on yield.

There is evidence that the negative relationship between RH and grain yield may be related to stomatal conductance (SC). In an environment with high humidity, the potential evaporation is generally lower and the consequential reduction in SC may result in a decreased photosynthetic rate [39, 40]. Furthermore, higher RH generally leads to higher risk of infection by fungal diseases [41, 42], which may additionally excerpt negative effects on wheat yields.

The observed challenge of identifying the impact of a single factor on crop yields due to confounding effects is confirmed by several previous studies [36, 43]. To overcome this challenge we employed crop model based scenario-analysis, which allowed us to separate the confounding effects and assess the isolated effect of changing a single weather factor, as also recommended by [10]. Incident GSR, as the factor most strongly influenced by API, was selected for the analysis. For both growing phases increasing GSR led to increasing yield and decreasing GSR led to decreasing yield, with the effects being stronger for the late compared to the early growing phase. It is likely that changes in radiation are more effective during the late phase, as the greater part of biomass growth and photosynthetic activity occurs after winter recovery [44]. The results furthermore showed that for all growth phases the negative yield effects of reduced radiation were stronger, than the positive yield effects of increased radiation. With the incident GSR continuously decreasing in China over the last decades [12], it is very likely that wheat production in the NCP is already situated at the lower end of required radiation. Any further increase of air pollution and consequential reduction in incident GSR may act strongly negative on wheat yields. Accordingly, [26] and [27] identified decreasing wheat yields in consequence of reduced radiation.

However, it also needs to be considered, that APSIM-Wheat does so far not account for changes in the ratio of direct and diffuse radiation, but just runs with total GSR as radiation input [27, 45]. This may lead to certain overestimations of the negative effects of air pollution induced radiation reductions on crop yields. The reason is that increasing air pollution leads to an increasing fraction of diffuse radiation in total radiation [35, 46, 47, 48], which significantly increases the light availability and hence photosynthesis in the shaded (and normally light deficient) part of the crop canopy [49]. This positive effect generally overcompensates for the negative photosynthetic effect in the sunlit (and normally light saturated) part of the canopy, which is caused by air pollution induced reduction in direct radiation [50]. In China, where optically-scattering aerosols, such as sulfate, organic carbon, nitrate, ammonium and mineral aerosols are dominant [51], high air pollution conditions are reported to increase the share of diffuse radiation to more than 60% of total radiation [47].

With regard to changed solar radiation composition, reduced UVR in consequence of high air pollution may also be beneficial for early growth of winter wheat. Air pollution is often associated with ozone depletion [52], with negative consequences for plants, since biologically active short-wavelength ultraviolet-B radiation increases under reduced ozone concentrations [53]. Accordingly, our regression analysis revealed a positive, though insignificant, relationship of reduced UVR and wheat yield.

The above described potential benefits of air pollution induced changes in the radiative properties of solar insolation on crop growth, which may partly compensate for some of the negative effects of reduction in total GSR, can so far not be simulated with APSIM-Wheat. Hence to better capture effects of air pollution on wheat growth and yield future improvements of APSIM should include the consideration of radiation properties beyond sole GSR. As measured data on the shares of diffuse and direct radiation are generally rare, a module should be integrated in APSIM that estimates the shares of both radiation components based on measured total radiation, latitude and day of year, e.g., following [54, 55]. Then the effects of changes in the shares of direct and diffuse radiation on canopy assimilation need to be simulated following, e.g., [50, 56].
